# Differential Modulation of TREM2 Protein during Postnatal Brain Development in Mice

**DOI:** 10.1371/journal.pone.0072083

**Published:** 2013-08-19

**Authors:** Mariela Chertoff, Kalpana Shrivastava, Berta Gonzalez, Laia Acarin, Lydia Giménez-Llort

**Affiliations:** 1 Department of Cell Biology, Physiology and Immunology, Universitat Autonoma Barcelona, Barcelona, Spain; 2 Department of Psychiatry and Forensic Medicine, Universitat Autonoma Barcelona, Barcelona, Spain; 3 Institute of Neuroscience, Universitat Autonoma Barcelona, Barcelona, Spain; Virginia Commonwealth University, United States of America

## Abstract

During postnatal development, microglia, the resident innate immune cells of the central nervous system are constantly monitoring the brain parenchyma, cleaning the cell debris, the synaptic contacts overproduced and also maintaining the brain homeostasis. In this context, the postnatal microglia need some control over the innate immune response. One such molecule recently described to be involved in modulation of immune response is TREM2 (triggering receptor expressed on myeloid cells 2). Although some studies have observed TREM2 mRNA in postnatal brain, the regional pattern of the TREM2 protein has not been described. We therefore characterized the distribution of TREM2 protein in mice brain from Postnatal day (P) 1 to 14 by immunostaining. In our study, TREM2 protein was expressed only in microglia/macrophages and is developmentally downregulated in a region-dependent manner. Its expression persisted in white matter, mainly in caudal corpus callosum, and the neurogenic subventricular zone for a longer time than in grey matter. Additionally, the phenotypes of the TREM2+ microglia also differ; expressing CD16/32, MHCII and CD86 (antigen presentation markers) and CD68 (phagocytic marker) in different regions as well as with different intensity till P7. The mannose receptor (CD206) colocalized with TREM2 only at P1–P3 in the subventricular zone and cingulum, while others persisted at low intensities till P7. Furthermore, the spatiotemporal expression pattern and characterization of TREM2 indicate towards its other plausible roles in phagocytosis, progenitor’s fate determination or microglia phenotype modulation during postnatal development. Hence, the increase of TREM2 observed in pathologies may recapitulate their function during postnatal development, as a better understanding of this period may open new pathway for future therapies.

## Introduction

Microglia are the resident innate immune cells of the central nervous system (CNS), ontogenically related to macrophages and they derive from yolk sac to enter brain at early embryonic development [1], [2]; migrating through the brain following a specific pattern [3]. While vascularization progresses, they locally proliferate in the ‘fountains of microglia’ and populate all the parenchyma during the first two weeks of life [4], [5], [6], [7], [8]. Concomitantly, the postnatal development comprises neuronal and glial differentiation, dendritic arborization, axonal growth, myelination, blood-brain barrier formation and neuronal circuits’ establishment, including new synaptic contacts formation. Microglia constantly survey the parenchyma [9], [10], clean the cell debris or synaptic contacts overproduced during postnatal development and play an important role in maintaining the homeostasis [11], [12], [13]. In the ongoing process, microglia display distinct physiological and morphological features [7], [14]; that indeed is due to microglia being endowed with a plethora of molecules that allow them to acquire diverse and complex phenotypes [15], [16], [17], [18], [19].

The postnatal microglia need to control the innate immune response in context to cellular death during development using endogenous mechanisms regulating inflammatory cell activation, including the expression of modulatory and/or inhibitory membrane receptors. These receptors play a key role in the regulation of inflammatory processes, mainly by cell-cell interaction with neurons, such as CX3CR1-CX3CL1 [20], CD200R-CD200 [21], [22], [23], CD47-CD172a [24] and TREM2-HSP60 (as putative endogenous receptor) [25], [26], [27], among others.

TREM2 (triggering receptor expressed on myeloid cells 2) is a cell surface receptor expressed on osteoclast, dendritic cells, macrophages, nature killers, neutrophils and microglia [26], [28], [29], [30], [31]. Recently the expression of TREM2 in subpopulation of neurons and oligodendrocytes has been described [31], [32], [33]. TREM2 needs an adaptor protein DAP12 to initiate the intracellular signalling cascade via an immunoreceptor tyrosine-based activation motif (ITAM) domain and tyrosine-kinases [28]. The complex TREM2/DAP12 had also been associated with synaptic function [34], [35]. In humans, the loss-of-function of TREM2 or its coreceptor DAP12 is responsible for the recessively inherited Nasu-Hakola disease (also known as *Polycystic lipomembranous osteodysplasia with sclerosing leukoencephalopathy;* PLOSL), characterized by an early onset dementia associated with bone cysts [36], [37]. The brains of PLOSL affected patients show strong microglial activation in the cerebral white matter, substantial white matter atrophy in corpus callosum and basal ganglia [36]. In addition, failure of TREM2 function in microglia has been associated with the loss of homeostatic control [38], [39].

Studies *in vitro*, using macrophages have shown that TREM2 is down-regulated by inflammatory stimulus such as LPS [26] and is induced by anti-inflammatory cytokine IL-4 [40]. Additionally, TREM2 stimulation by lentiviral transgene expression in microglial cells induced cytoskeletal reorganization and increased phagocytosis, whereas knockdown of TREM2 induced a reduced phagocytic ability and increased capacity to produce pro-inflammatory molecules like TNF-alpha and iNOS [41]. Experiments defining TREM2 function after CNS pathology have shown that blockade of TREM2 in animal models of multiple sclerosis leads to disease exacerbation by increasing leukocyte infiltration and demyelination [42], while intravenous application of TREM2-transduced myeloid cells facilitated repair by reducing axonal damage [43]. Interestingly, recent experiments using a mouse stroke model had shown an attenuated inflammatory response in knock out animals compared to wild type [44], without changes in injury size.

During the last few years, some groups have described the TREM2 mRNA expression in embryonic and postnatal brain [27], [33]. However, the regional pattern and phenotype of cells expressing functional TREM2-DAP12 during postnatal development is still exiguous or missing. Moreover, the type of cells expressing TREM2 is quite debatable. Hence, the aims of this study was to analyze the regional and age dependent expression pattern of TREM2 protein in postnatal brain by immunofluorescence. An emphasis was placed in the characterization of the phenotype of the cell population expressing TREM2. The temporal and regional modulation of TREM2 might give us the basis for a better understanding of mechanisms involved in neuropsychiatric and neurodegenerative disorders following perinatal brain injury or inflammation in future.

## Materials and Methods

### Animals

C57BL/6 mice (breaded in Harlan Labs, France) of different postnatal ages were used for all the experiments. Animals were maintained at constant temperature (24±2°C) and housed on a 12∶12 hours light-dark cycle with food and water *ad libitum*. Experimental animal work was conducted in accordance with Spanish regulations and the European Union directives on the use of animals in scientific research. The protocol was specifically approved by the Ethical Commission for Animal and Human Experimentation of Autonomous University of Barcelona (protocol number 811). All efforts were made to minimize number and suffering of animals used at each stage of experimentation.

### Tissue Processing

C57BL/6 mice were sacrificed at postnatal day (P) 1, P3, P5, P7, P10, P12 and P14. Animals from at least 3 different litters were intraperitoneally anaesthetized (ketamine and xylacine 80/10 mg/Kg) and intracardially perfused using 4% paraformaldehyde in phosphate buffer saline (PBS, pH 7.4). Subsequently, brains were removed, postfixed for 4 hrs in the same fixative, cryoprotected in 30% sucrose, frozen with dry CO_2_, and finally stored at −80°C until use. Brains were serially cut in a cryostat (Leica CM3050 S) into 30 µm thick coronal sections and stored mounted on Flex IHC slides (K8020, Dako) at −20°C.

### Immunohistochemistry for TREM2

Animals from each postnatal age group (n = 3–5) were processed for the immunohistochemical demonstration of TREM2. Single immunohistochemistry was initiated by blocking the endogenous peroxidase (2% H_2_O_2_ in 70% methanol for 10 min) and incubation for 1 h in blocking buffer (BB) containing 0.2% gelatine (powder food grade, 1.04078, Merck) in Tris-buffered saline (TBS, pH 7.4) with 0.3% Triton X-100 (TBST) at room temperature (RT). Afterwards, slides were incubated overnight at RT with the primary antibody diluted in BB (**Table 1**) and the slides used as negative controls without primary antibody. In a separate slide, TREM2 primary antibody was pre-blocked with TREM2-Fc protein, as recommended by supplier (25x, 1729-T2, R&D System) in order to ensure the specificity (no staining was observed in these conditions). After washing with TBST, sections were incubated with biotinylated anti-sheep antibody at RT for 1 h (**Table 2**); washed again and incubated with streptavidin–peroxidase for 1 h (**Table 2**). The peroxidase reaction was visualized by incubating the sections in 3,3-diaminobenzidine and hydrogen peroxide using the DAB kit (SK-4100; Vector Laboratories, USA). Finally, sections were dehydrated and coverslipped in DPX. Photography was performed using a DXM 1200F Nikon digital camera joined to a Nikon Eclipse 80i microscope and plates were arranged using Adobe Photoshop CS3.

**Table 1:** List of primary antibodies used for immunostaining.

Target antigen	Host	Dilution	Catalogue Number, Brand
TREM-2	Sheep	1∶400, 1∶300 (IHC, IF)	AF1729, R&D
GFAP	Rabbit	1∶750	Z0334, DAKO
Iba-1	Rabbit	1∶500	019-19741, DAKO
DAP12	Rabbit	1∶100	AB4070, Millipore
Tomato lectin	Biotinylated	1∶150	L0651, Sigma
PDGFRalpha	Rat	1∶1000	558774, BD Pharmigen
Olig2	Rabbit	1∶200	18953, IBL
CD68	Rat	1∶1000	MCA1957,AbD Serotec
CD86	Rat	1∶200	550542, BD Pharmigen
CD206	Rat	1∶500	MCA2235,AbD Serotec
MHCII	Rat hybridoma	1∶25	TBI-120, ATCC
CD16/32	Rat	1∶500	553142, BD Pharmigen

IHC: immunohistochemistry.

IF: immunofluorescence.

**Table 2:** List of secondary antibodies used for immunostaining.

Conjugate	Host	Dilution	Catalogue Number,Brand
Biotinylated	Anti-sheep	1∶500	BA6000, Vector
Alexa 488	Anti-sheep	1∶500	A11015, Invitrogen
Alexa 568	Anti-rabbit	1∶500	A10042, Invitrogen
Cy5	Anti-rabbit	1∶500	A10523, Invitrogen
Alexa 594	Anti-rat	1∶500	A21209, Invitrogen
Streptavidine - Cy5		1∶500	PA45001, AmershamBiosciences
Streptavidine - HRP		1∶500	SA-5004, Vector

### Quantification of TREM2 in Postnatal Brain

**Figure 1:**
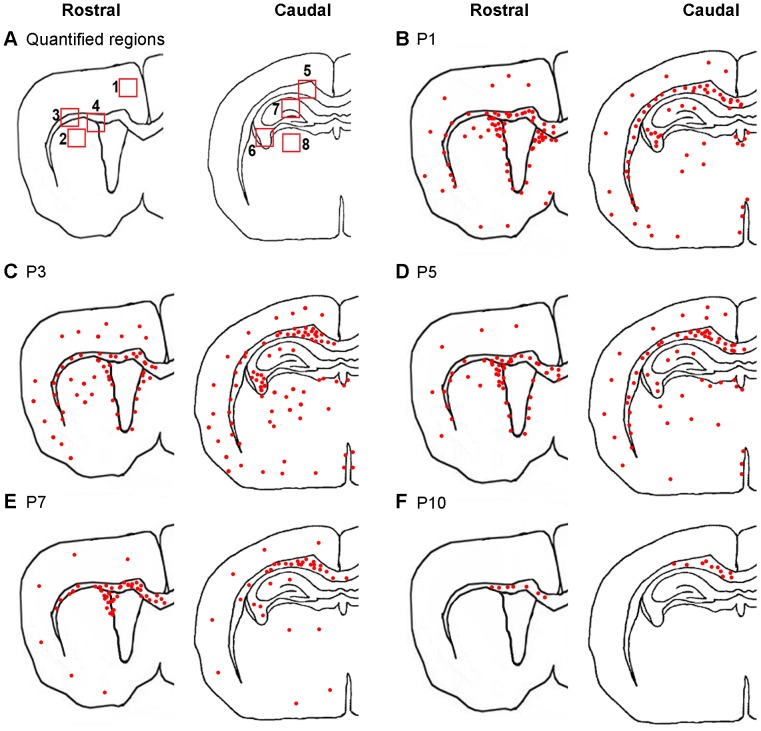
Distribution of TREM2 protein during postnatal development. A). Schemes showing the regions quantified for TREM2 by immunohistochemistry**:** 1-Cortex; 2-Caudate-Putamen; 3-Rostral Corpus callosum; 4-subventricular zone; 5-Caudal corpus callosum; 6-fimbria; 7-hippocampal fissure and 8-thalamus. (B-F) Patterns of TREM2 expression were depicted as red dots in schemes showing rostral and caudal region of postnatal brains at P1 (B), P3 (C), P5 (D), P7 (E) and P10 (F).

Images from three sections/animal were taken, representing the following regions: cingulate cortex (cx), subventricular zone (svz), rostral and caudal corpus callosum (cc), caudate-putamen (cp), hippocampal fissure (hf), fimbria (fim) and thalamus (tl) (Fig. 1A). Micrographs were captured using the 40× objective. For P1, P3 and P5 photographs were taken from 2.43 to 2.79 mm and 3.39 to 3.75 mm (corresponding to Fig 57–60 and 65–68, respectively in the Atlas of the Developing Mouse Brain [45]). From P7 onwards regions were chosen as follows: from 3.03 to 3.27 mm and from 4.47 to 4.71 mm (corresponding to Fig 117–119 and 129–131, respectively in the Atlas of the Developing Mouse Brain [45]). ImageJ software (National Institutes of Health) was used for unbiased quantitative analysis of immunoreactive TREM2+ area/mm^2^, using a method previously described [46]. Data were shown as TREM2+ area/mm^2^± S.E.M.

### Colocalization Studies

Double or triple immunofluorescent staining was used for the characterization of cells expressing TREM2. At least three animals from different litters were used from each immunostaining at all ages. Briefly, sections on slides were washed with TBS-T and incubated in BB at RT for 1 hr followed by overnight incubation at RT with primary antibodies prepared in BB (**Table 1**). Samples were rinsed in TBST and incubated for 1 hr at RT with corresponding secondary antibodies (**Table 2**). For tomato lectin (TL) labeling, samples were washed after incubation with primary antibodies and subjected to incubation with biotinylated-TL for 2 h at 37°C. This was followed by incubation with streptavidin-conjugated secondary antibody. Before being coverslipped with Fluoromount G™ (0100-01, SouthernBiotech) the sections were rinsed and nuclei stained with 4′,6-diamidino-2-phenylindole (DAPI,1∶10000, D9542, Sigma Aldrich). Colocalization was analyzed with confocal microscope D1 Axio Examiner LSM 700 (Zeiss). Final plates were composed using Adobe Photoshop CS3.

### Statistical Analysis

Data were tested for normality (Shapiro–Wilks test) and variance homogeneity (Levene test). Comparisons were performed using ANOVA followed by Tukeýs test or Kruskal Wallis followed by multiple comparison test, where appropriate (Infostat Version 2013, InfoStat Group, URL:http://www.infostat.com.ar). Significance was recorded at p<0.05.

## Results

### Postnatal Distributions of TREM2 Show an Age-dependant Regional Pattern

We first studied the TREM2 distribution during postnatal development using immunohistochemistry, which is summarized in schemes with red dots (Fig. **1B–F**). A high expression of TREM2 was not only observed in subventricular zone (svz) and neuroephitelium but also in white matter regions, especially in corpus callosum (cc), external capsule and fimbria. Developmental downregulation of TREM2 was observed, showing a faster reduction in grey matter than in white matter, where it was detectable till P10, almost exclusively in cc (Fig. **1F**). TREM2 was undetectable by immunohistochemistry after P10 at all regions studied (data not shown) hence rest of the studies were carried out till P10.

### Quantification of Developmental Changes on TREM2+ Area

Quantification of TREM2+ area was performed in regions were qualitative changes were observed and areas were the absence of TREM2 had been previously correlated with pathologies. TREM2+ area in eight regions were measured as shown in Fig. **1A** **.** The TREM2+ areas/mm^2^ were measured using ImageJ software and are detailed below.

#### Subventricular zone and Neuroephitelium

**Figure 2:**
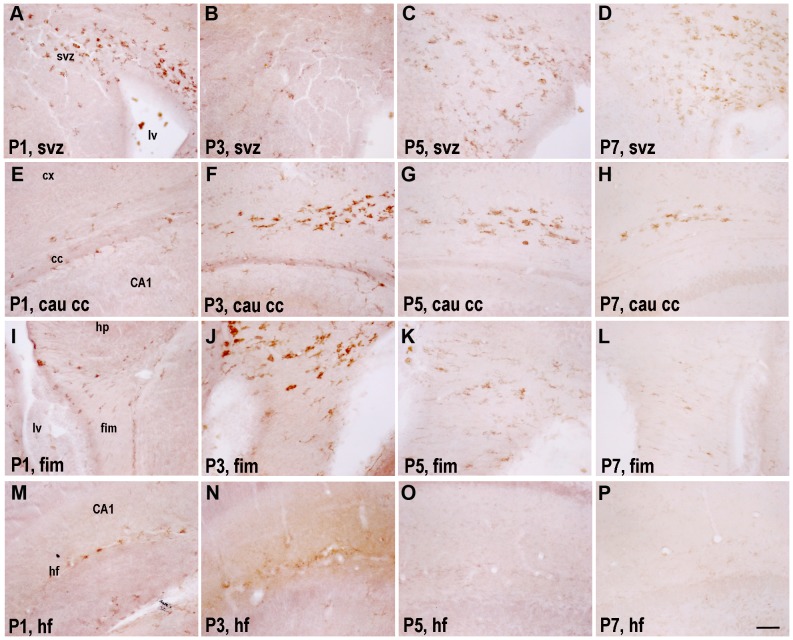
TREM2 immunostaining showing developmental modulation. (A–D) Developmental changes in subventricular zone (svz) at P1 (A), P3 (B), P5 (C) and P7 (D), showing a reduction in TREM2 expression at P3 and then an increase from P5 to P7. (E–H) Developmental changes in caudal corpus callosum (cau cc) at P1 (E), P3 (F), P5 (G) and P7 (H), showing maximum TREM2 expression at P3 followed by a progressive reduction till P7. (I–L) Changes in fimbria (fim) at P1 (I), P3 (J), P5 (K) and P7 (L), with a maximum expression at P3. (M–P) Developmental expression of TREM2 in hippocampal fissure (hf) at P1 (M), P3 (N), P5 (O) and P7 (P), showing no modulation of TREM2. lv: lateral ventricle; hp: hippocampus; CA1:cornu ammonis 1. Scale bar = 50 µm.

**Figure 3:**
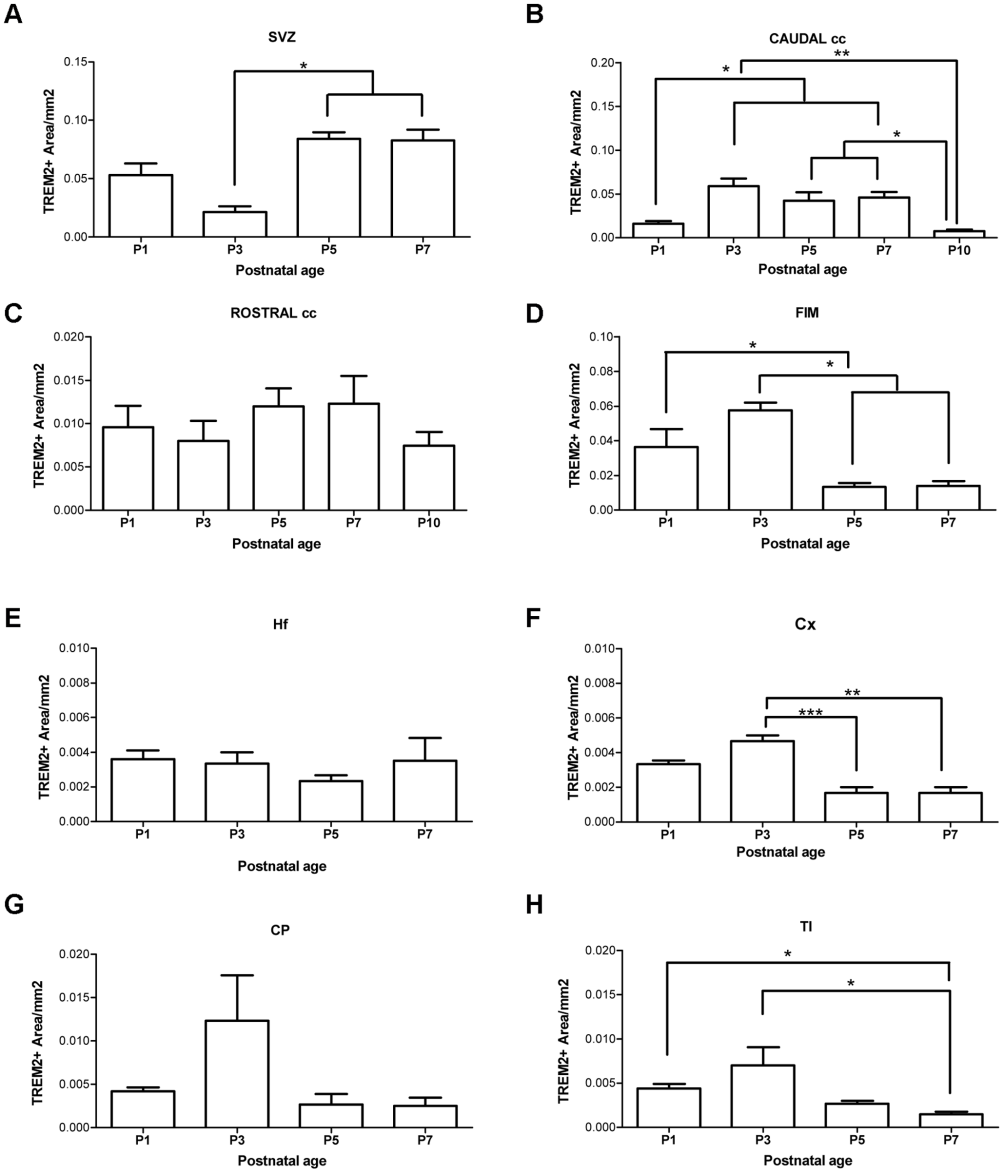
TREM2 expression was differentially modulated in a region and age dependent manner. Quantification of TREM2+ area/mm^2^ in subventricular zone, SVZ (A); caudal corpus callosum, cc (B); rostral Corpus callosum (C); fimbria, FIM (D); hippocampal fissure, Hf (E); Cortex, Cx (F); Caudate Putamen, CP (G) and thalamus, Tl (H). Three to five animals were used at each time point. Data were represented as mean ± SEM. Analysis was performed using one-way ANOVA followed by Tukey *post hoc* test or Kruskal Wallis followed by multiple comparison tests as suitable. *p<0.05; **p<0.01; ***p<0.001.

These regions expressed the highest level of TREM2 compared to other regions analyzed in this study. Interestingly, the TREM2+ area is quite small at P3 compared to later time points studied, showing significant differences from P5 and P7 (Fig. **2A–D** and **3A**). The expression of TREM2 in svz at P10 was undetectable (data not shown). The morphology of TREM2+ cells was mainly round and amoeboid in this region at P1 (Fig. **2A**), progressing to primitive ramified form at P7 (Fig. **2D**).

#### Corpus callosum

The TREM2+ area in caudal cc increased at P3–P7 and then decreased significantly after 10 days of birth, disappearing completely at later ages (Fig. **2E–H** and **3B**). These cells where mainly located at the edges between grey and white matter, moreover, the morphology varied from amoeboid to primitive ramified at all time points analyzed. TREM2 expression shows no modulation in the rostral cc (Fig. **S1A–D** and **3C**). Interestingly, the TREM2+ area in caudal cingulate cc was higher than in the rostral cc (Fig. **2E–H**, S**1A–D**, **3B–C**); indeed depicting developmental modulation.

#### Fimbria

The TREM2+ area showed a peak of expression at P3 (Fig. **2I–J** and **3D**), diminishing later until completely disappearing at P10 (Fig. **2K–L** and data not shown). The expression is mainly concentrated in the upper part of the fimbria, adjoining CA3 region of hippocampus. The morphology varied with age as amoeboid and primitive ramified cells were present at P1 through P5 (Fig. **2I–L** **)**. At P7, only primitive ramified and ramified morphology were found.

#### Hippocampal fissure (hf)

TREM2 expression was restricted to hf of the medial and ventral hippocampus without any developmental modulation (Fig. **1**, **2M–P** and **3E**). At P1, TREM2 was expressed by some microglia/macrophages inside and around the blood vessels of hf; however, their expression diminished with age (Fig. **2M–P**).

#### Cortex and Caudate-Putamen (cp)

At P1, scattered expression of TREM2 was observed with the cells being very dispersed and limited to layers III to VI of the cortex and to dorsal region of cp (Fig. **S1E** and **S1I**). The TREM2+ area in cortex was maximum at P3, showing significant differences with respect to later time-points analyzed (Fig. **S1F–H and Fig3F**). The morphology of TREM2+ cells changed from primitive ramified at P1 to ramified at P7, accompanied by a reduction in TREM2 intensity (Fig. **S1E–L**). No modulation of TREM2 expression was observed in cp during postnatal development (Fig. **S1I–L** and Fig **3G**).

#### Thalamus

As in other grey matter regions, TREM2+ area increased from P1 to P3 and then slowly disappeared at P10 (Fig. **1**, **S1M–P**). Significant differences appeared between P1–P3 and P7 mice in this region (Fig. **3H**). Although, amoeboid TREM2+ cells persisted at each time point analyzed, primitive ramified cells appeared from P1 to P5 (Fig. **S1M–O**).

### Postnatal Brain Expressed Functional TREM2

**Figure 4:**
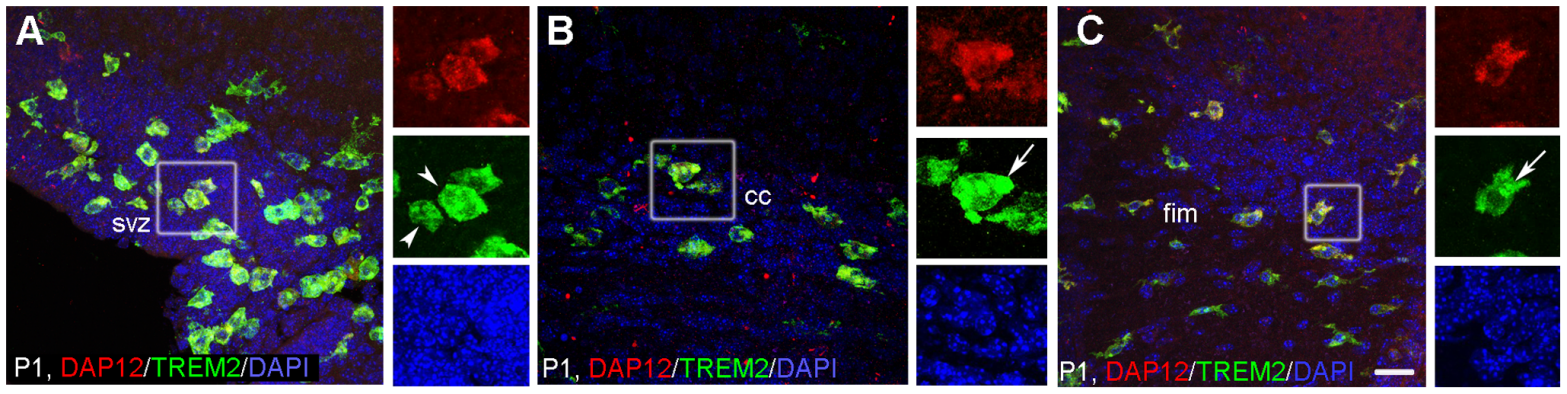
Expression of functional TREM2–DAP12 cells in postnatal brain. Immunofluorescence study of TREM2 (green), coreceptor DAP12 (red), and nuclear stain DAPI (blue) was performed. Colocalization of TREM2 and DAP12 was found in subventricular zone, SVZ (A), corpus callosum, cc (B) and fimbria, fim (C). “Foamy” cells (arrowheads) and intracellular aggregation of TREM2 (arrows) was also observed. Colocalization can be seen in yellow. Scale bar A–C = 20 µm.

In order to determine the functional state of TREM2, we analyzed the expression of DAP12 and its colocalization with TREM2+ cells in postnatal brain from P1 to P7. Confocal analysis demonstrated that almost all TREM2+ cells colocalized with DAP12 (Fig. **4A–C**) in each region and time-point studied, especially at P1 when the highest intensity of DAP12 expression was observed. The presence of “foamy cells” (arrowheads in Fig. **4A**) suggests phagocytic activity at this time-point. In addition to membranous expression, we found that TREM2 was also intracellularly located in some cells (arrows in Fig. **4B–C**).

### Lineage of TREM2+ Cells

**Figure 5:**
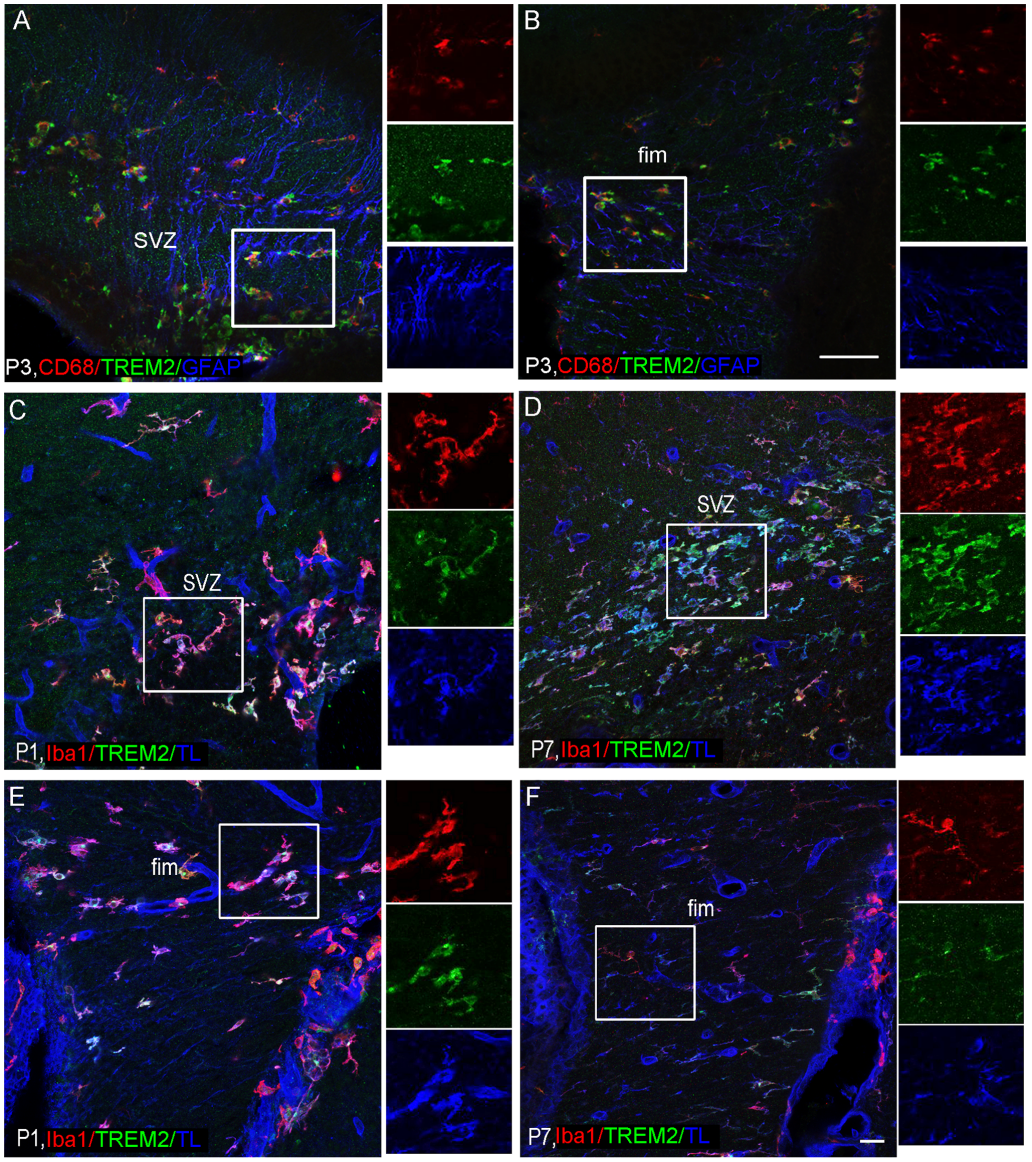
TREM2 expression was found on microglia. Triple immunofluorescence was performed to characterize the population of TREM2+ cells. (A–B) TREM2 colocalized with CD68+ cells, a marker of phagocytic activity but not with radial glia or astrocytes (GFAP+ cells) at P3 in subventricular zone, SVZ (A) and fimbria, fim (B). Insets beside the figures show separated channels: CD68 in red, TREM2 in green and GFAP in blue. (C–F) TREM2+ cells colocalized with Iba-1+ cells but not with blood vessels. At P1, all Iba-1+ cells expressed TREM2 in SVZ (C) and fimbria (E). The expression of TREM2 was restricted to a subpopulation of microglia at P7 (D and F). Insets in C–F represent separated channels: Iba1 in red, TREM2 in green and Tomato lectin (TL) in blue. Triple colocalization can be seen in purple. Scale bar A–B = 50 µm; C–F = 20 µm.

To characterize cells expressing TREM2 protein during postnatal development, we performed double/triple immunofluorescence for markers of microglia (Iba-1, CD68, TL), oligodendrocyte (olig2 and PDGFRalpha), astrocytes (GFAP) and endothelial cells (TL). Confocal analysis for colocalization demonstrated that TREM2 protein was expressed by microglia/macrophages during normal postnatal development (Fig. **5**). We did not find any colocalization with oligodendrocyes (Fig. S2), astrocytes (Fig. **5A–B**) or endothelial cells (Fig. **5C–F**). No noticeable association of TREM2+ cells with the radial glia (Fig. **5A–B**) or blood vessels (Fig. **5C–F**) was observed. A marker for phagocytic activity (CD68) was expressed throughout the parenchyma, but only a subpopulation of CD68+ cells colocalized with TREM2.

As brain developed, there was an increase in number of microglia although a decrease in TREM2 expression was observed. It is noteworthy that all TREM2+ cells were microglia/macrophages in each region/time analyzed (Fig. **5A–F**). Nevertheless, the Iba1+/TREM2+ cells were consistently reduced in grey matter at P7 along with a decrease in CD68+/TREM2+ cells.

### Phenotypic Characterization of TREM2+ Cells

With the purpose of analyzing the phenotypic features of postnatal TREM2+ cells, we studied the coexpression of TREM2 in cells expressing mannose receptor (CD206), Fc receptor (CD16/32) and antigen presentation markers (CD86 and MHCII).

#### Subventricular zone

**Figure 6:**
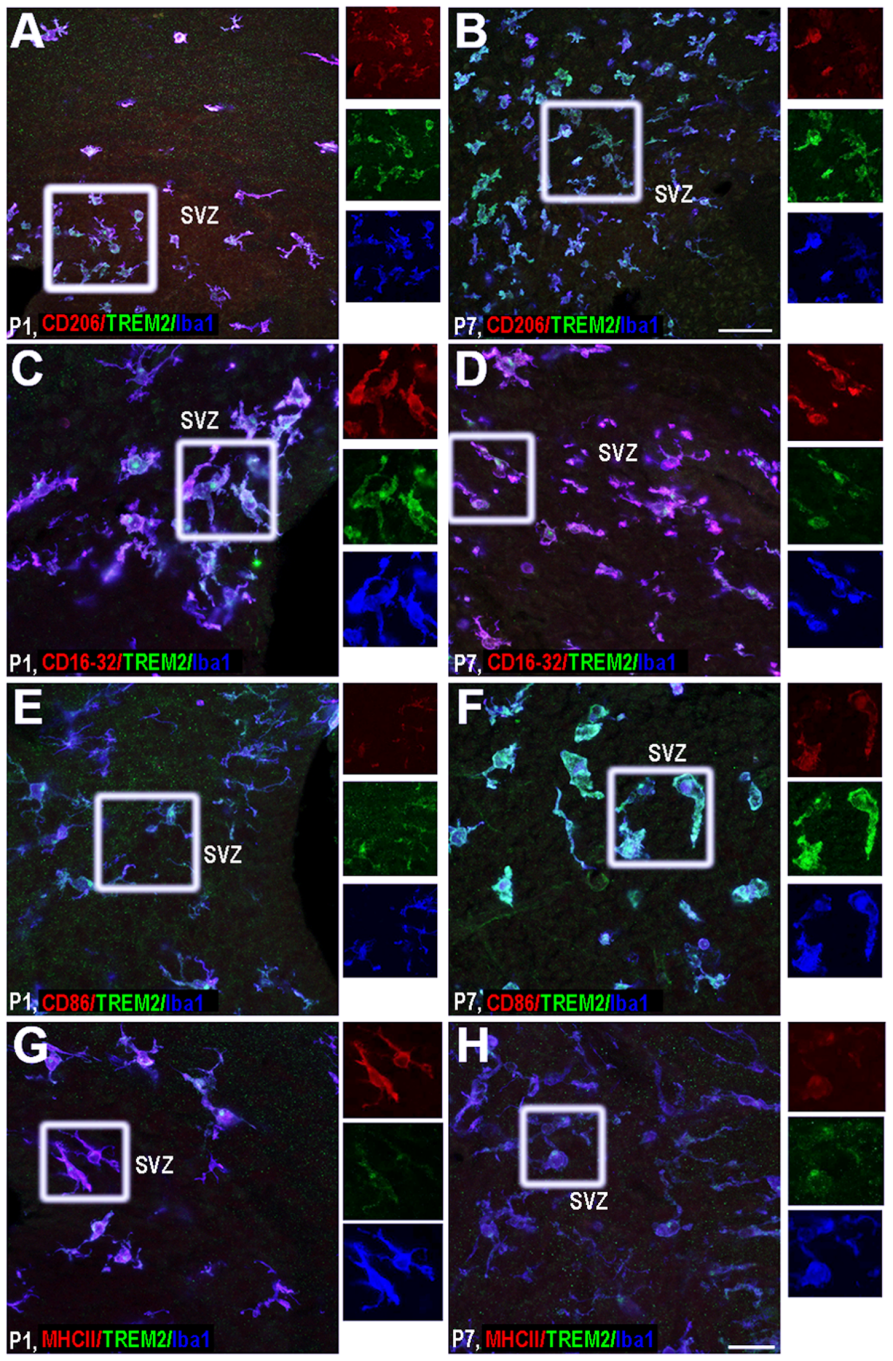
Phenotypic characterization of TREM2+ microglia in subventricular zone. TREM2 co-expression with: CD206 (A–B), CD16/32 (C–D), CD86 (E–F) and MHCII (G–H) was studied at P1 (A, C, E and G) and P7 (B, D, F and H). Insets beside each figure represent separated channels: CD206, CD16/32, CD86 and MHCII in red, TREM2 in green and Iba1 in blue. SVZ: subventricular zone. Triple colocalization can be seen in purple. Scale bar for A and B = 50 µm; scale bar for C–H = 20 µm.

The neurogenic niche showed specific features in microglia phenotype. Interestingly, only a subpopulation of TREM2+ cells expressed CD206 and CD86 (Fig. **6A, B, E and F**). Additionally, CD16/32 and MHCII expression in TREM2+ cells persisted during postnatal development in the svz, though at lower levels than in other regions at all ages studied (Fig. **6C, D, G and H**).

#### Cingulate cortex

At P1, the microglia were dispersed; in contrast to P7, when cells were found in all layers of the cortex, with processes covering almost all the parenchyma (data not shown). The colocalization of TREM2 with CD206 was observed in meningeal and perivascular TREM2+ macrophages (Fig. **S3A** and **B**); however, the intensity of staining on microglia was lower in the parenchyma and almost disappeared during postnatal development. CD16/32, CD86 and MHCII were also differentially regulated on TREM2+ cells during postnatal development. Although CD16/32 (Fig. **S3C** and **D**) and costimulatory molecule CD86 (Fig. **S3E** and **F**) were expressed at low levels in every time-point studied, the expression of MHCII on microglia was higher at P1 than at P7 (Fig. **S3G** and **H**). Noticeably, the expression of MHCII was not restricted to plasma membrane but appeared to be cytoplasmatic also (arrows in Fig. **S3H**).

#### Caudal corpus callosum

**Figure 7:**
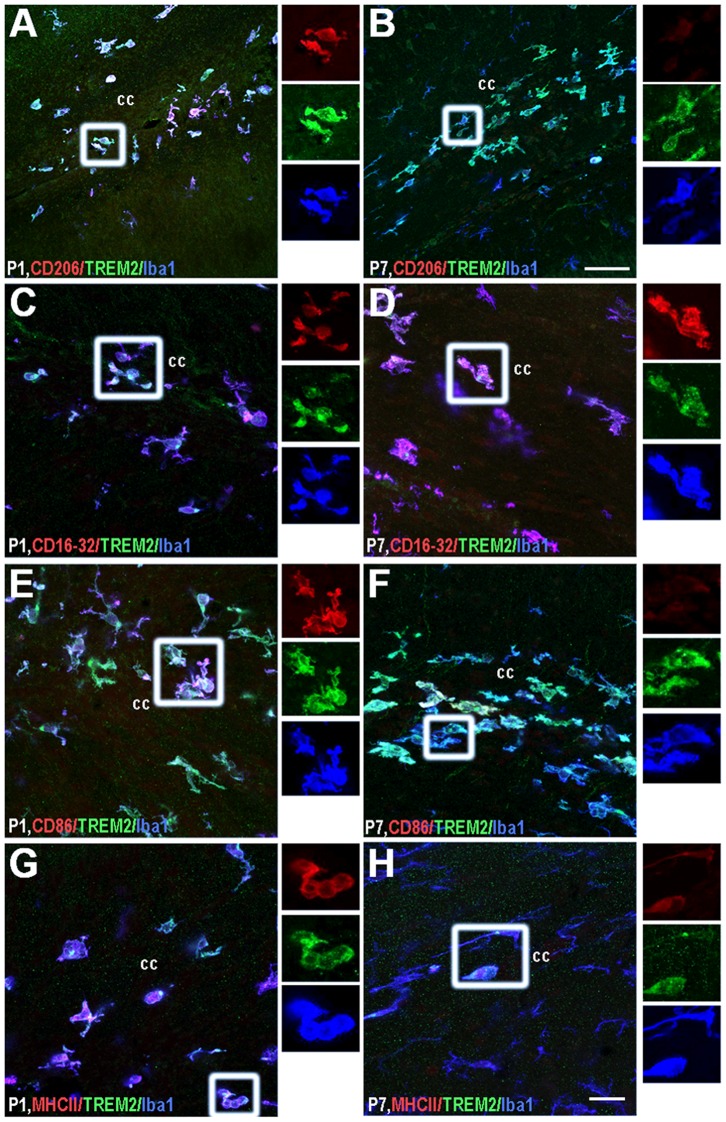
Phenotypic characterization of TREM2+ microglia in corpus callosum. TREM2 co-expression with: CD206 (A–B), CD16/32 (C–D), CD86 (E–F) and MHCII (G–H) was studied at P1 (A, C, E and G) and P7 (B, D, F and H). Insets beside each figure represent separated channels: CD206, CD16/32, CD86 and MHCII in red, TREM2 in green and Iba1 in blue. cc: corpus callosum. Triple colocalization can be seen in purple. Scale bar for A and B = 50 µm; scale bar for C–H = 20 µm.

The expression pattern of phenotypic markers on TREM2+ microglia were differentially regulated in cc during the first week of postnatal development. The TREM2+/CD206+ microglia were present here at P1 (Fig. **7A**). Although, some microglia were TREM2+ till P10 the expression of CD206 was lost with brain maturity (Fig. **7B** and data not shown). A similar scenario was observed in this region for CD86 (Fig. **7C and 7D**). Interestingly, expression of CD16/32 and MCHII remained constant in all TREM2+ microglia during the first week of life (Fig. **7E–H**).

#### Fimbria

At P1, microglia expressed all markers studied (Fig. **S4A, C, E** and **G**); however, they were independently modulated during postnatal development. Although expression of CD206 in parenchymal microglia was found at P1, the intensity reduced at P7 (Fig. **S4B**). It is noteworthy that CD206+/TREM2- cells were found inside the ventricles and ventricle linings at P7. Though the expression of CD16/32 was maintained in all microglia cells (Fig. **S4D**), CD86 was restricted to a subpopulation of microglia (Fig. **S4F**). The same pattern was observed for MHCII+ cells (Fig. **S4H**).

#### Hippocampal fissure

**Figure 8:**
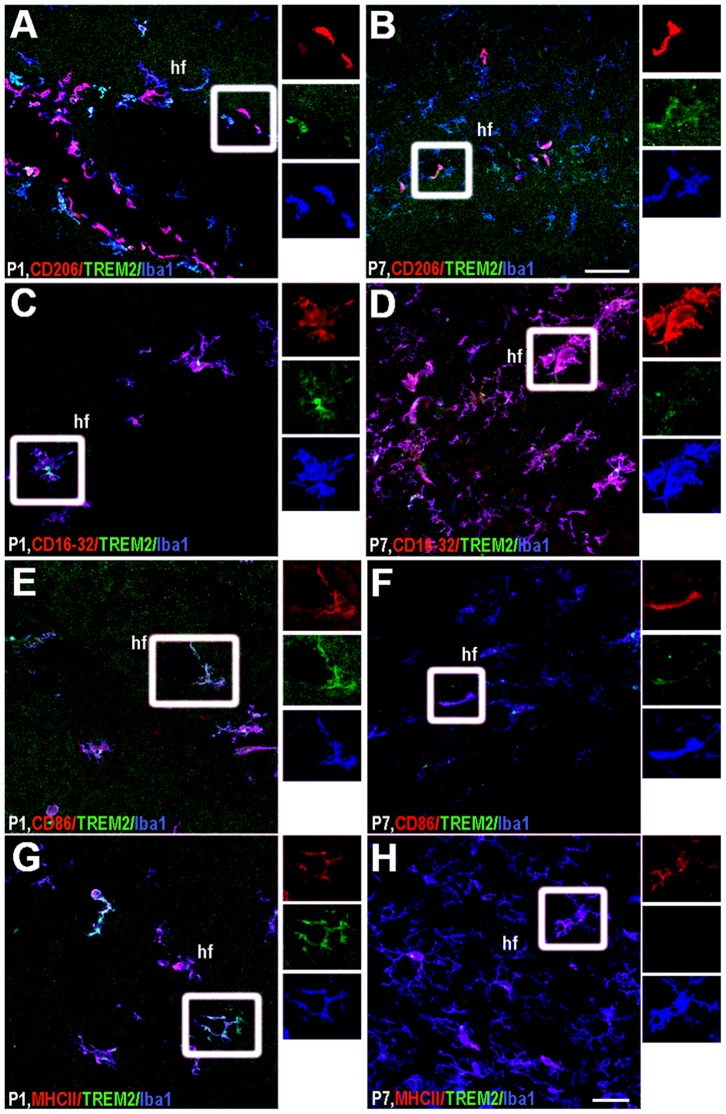
Phenotypic characterization of TREM2+ microglia in hippocampal fissure. TREM2 co-expression with: CD206 (A–B), CD16/32 (C–D), CD86 (E–F) and MHCII (G–H) was studied at P1 (A, C, E and G) and P7 (B, D, F and H). Insets beside each figure represent separated channels: CD206, CD16/32, CD86 and MHCII in red, TREM2 in green and Iba1 in blue. hf: hippocampal fissure. Triple colocalization can be seen in purple. Scale bar for A and B = 50 µm; C–H = 20 µm.

The expression of TREM2 was restricted to perivascular macrophages and some microglial cells at P1. The expression of CD206 was limited to perivascular macrophages and few of them colocalized with TREM2 at P1 (Fig. **8A**). TREM2+/CD206+ cells were not observed in the parenchyma at later timepoints (Fig. **8B**). Developmental modulation of CD16/32, CD86 and MHCII was also found in this region, where they co-expressed with TREM2+ microglia at P1. However, TREM2 co-expressed with a sub-population of CD86+ and CD16/32+ cells at later time points (Fig. **8C–F**). MHCII+TREM2+ cells were only present at P1 (Fig. **8G**) and P3 (data not shown), later being undetectable (Fig. **8H**).

**Figure 9:**
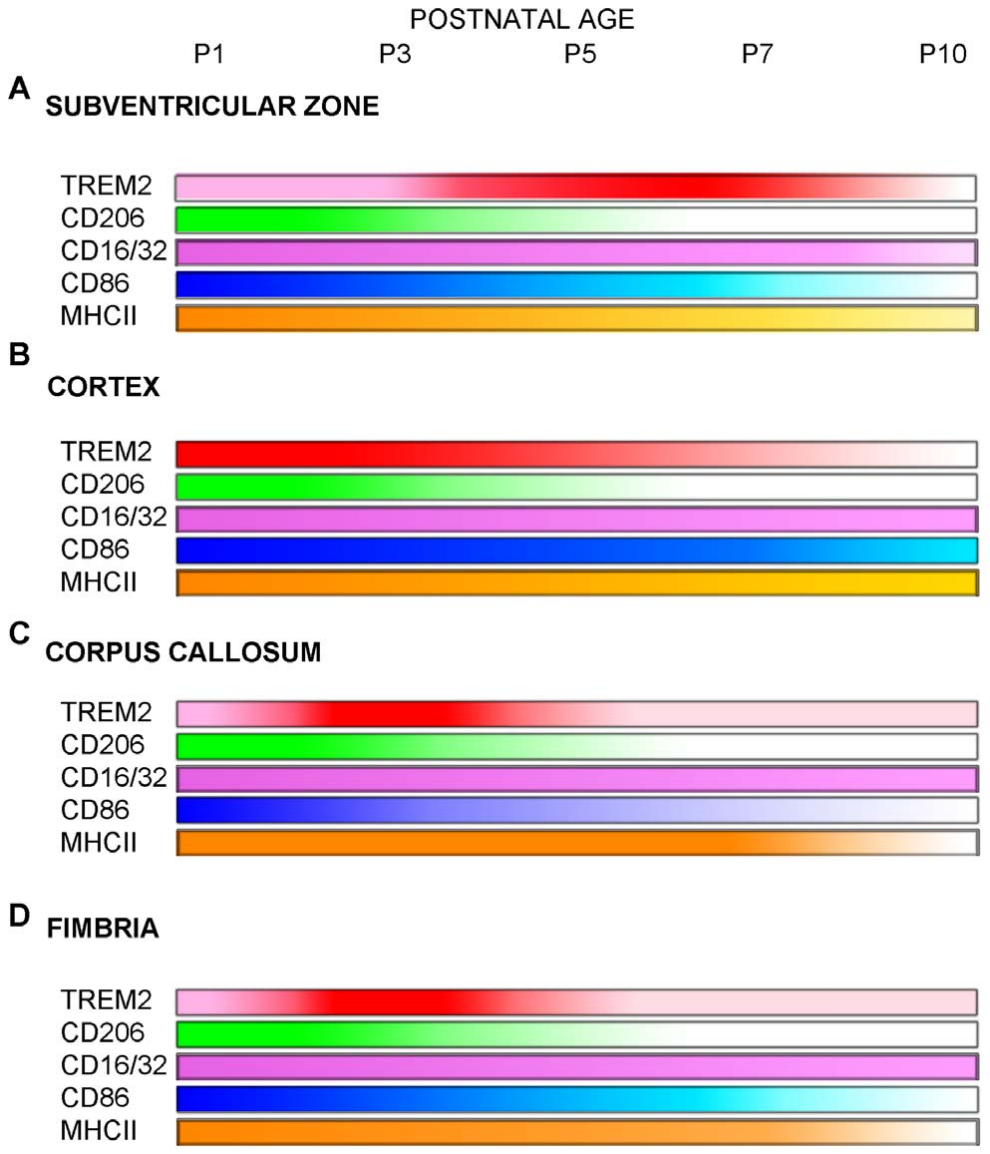
Schematic summary of spatio–temporal changes in TREM2 phenotypes. Developmental regulation of the TREM2+ phenotypes was shown in subventricular zone (A), cortex (B), corpus callosum (C) and fimbria (D) from P1 to P10. Regional changes in markers associated with TREM2 (red) where represent as color intensity: CD206 (green), CD16/32 (purple), CD86 (blue) and MHCII (orange).

To highlight the phenotypes of TREM2+ cells that changed in a region- and age-dependent manner, we summarized our findings in Figure 9, showing a qualitative description of these changes.

## Discussion

The goal of current study was to describe the regional and temporal expression of TREM2 protein during postnatal development as this is the first work of its type. Immunohistochemistry for demonstration of TREM2 allowed us to characterize regional phenotypes regulated by brain maturation. TREM2 was differentially regulated in grey matter and white matter during development. While TREM2 protein was not detected in grey matter after P5, it was still expressed in corpus callosum at P10. In previous studies from whole brain extracts, expression of TREM2 mRNA has shown little or no changes in their expression level from embryonic to adult brain [27], [33]. These discrepancies in results might be due to a post- transcriptional/translational regulation of TREM2 or the sensitivity of the technique used. Zhao and co-workers [47] had recently described the regulation of TREM2 by a NF-kB-sensitive miRNA as a mechanism of TREM2 control in Alzheimeŕs disease patients. Whether this system is functional during postnatal development needs to be studied.

To determine the phenotype of the TREM2+ cells during postnatal development double or triple immunofluorescence was performed. The study monitored colocalization of TREM2 with astrocytes, oligodendrocytes, endothelial cells or microglia so as to confirm previous work in adult brain [26]. In this study, all cells expressing TREM2 protein were microglia. Some reports have shown TREM2 expression on oligodendrocytes [27], [31], [32], [33]; although we have not seen any such colocalization in neonatal mice brain. Regional differences in TREM2 pattern were observed in grey and white matter during postnatal development in concordance with previous study in adults analyzing TREM2 mRNA [26].

Our results show DAP12 (coreceptor for TREM2) expression in almost all TREM2+ cells analyzed suggesting a complete system for functional signals. In accordance with our results, DAP12 was previously described as being restricted to microglia in postnatal CNS [34], [35]. Similar to previous *in vitro* report in microglia [48] showing two different pools of TREM2 that shuttled to and from plasma membrane.

TREM2-DAP12 coexpression and TREM2+CD68+ ‘foamy’ microglia/macrophages were observed throughout development in the svz, the region with higher expression of TREM2 in our study. Previous studies report that TREM2 expression increase after injury induced cell death in adults [43], [44], [49] and that DAP12 is involved in neuronal apoptosis during development [34]. Moreover, the interaction of progenitors with astrocytes and microglia in svz leads to progenitor fate recognition [50], [51]. Our findings suggest the putative role of TREM2-DAP12 in clearance of overproduction of cells and cell debris. Whether this complex plays a role in the fate of progenitors in the svz needs further elucidation.

During the first week of life, the microglia transform from round/amoeboid to a ramified morphology while reaching their final destination. We observed that the round or amoeboid TREM2+ microglia progressively disappeared in grey matter as the TREM2- ramified morphology was acquired. Since ligands for TREM2 has been described on astrocytes [25] and some reports also suggest that astrocytes play an important role in determining the mature microglial phenotype [52], [53], [54]. We hypothetized that signals derived via TREM2 ligand-receptor interactions might modulate the microglial phenotype in neonatal brain.

The morphological differentiation of microglia/macrophages is usually accompanied by changes in their immunophenotype [55], [56], which reflect the functional state of microglia. Although the classification of these phenotypes has been extensively described in the adult CNS, the spatio-temporal regulation of postnatal microglia has been partially understood [8], [57], [58]. Unlike other myeloid cells, postnatal microglia remain in an undifferentiated state [59]; hence, to decipher the involvement of TREM2 during maturation we analyzed various microglial markers. Regional differences on microglia phenotype in healthy [17], [60], [61] and injured brain has been thoroughly investigated [62], [63], [64]; but there is scarcity of data regarding TREM2+ microglia in neonates. We choose to monitor molecules as CD68, MHCII and CD86 usually associated with pathologies in TREM2+ microglia/macrophages during normal development to deduce if they are playing a part in the active phagocytosis happening during the first week of life when TREM2 expression was notable.

TREM2 is upregulated by IL-4 [40] and has been proposed as a marker for protective function of microglia. Also, IL-4-activated microglia have been shown to be pro-neurogenic [62], [65] and neuroprotective [58]. IL-4 is expressed in several regions of postnatal brain [66] and stimulates alternative activation markers such as CD206 [58]. In this regard, we have observed a region- and age-dependent modulation of TREM2+CD206+ cells during early (P1–P3) postnatal development possibly affecting excessive apoptosis at this stage. Our results suggest that TREM2+ postnatal microglia are endowed with more of an anti-inflammatory/protective phenotype (CD206+).

In accordance with our observation, it has been shown that the expression of MHCII on microglia in the parenchyma appears both cytoplasmatic and associated with membrane [67]. Additionally, it has been demonstrated that TREM2 increased the expression of MHCII and CD86 in dendritic cells [30] but no changes have been observed on cultured microglia overexpressing TREM2 [41]. Our observation that these markers in microglia were downregulated faster in grey matter than in white matter, correlates with the study by Dalmau and colleagues [7] showing microglia attaining maturity earlier in grey matter. Throughout the first week when microglia are distributing in the parenchyma and actively performing apoptosis, some TREM2+ cells also express MHCII and CD86 in specific regions. Thus, we may assume that this change of phenotype and typical regional distribution shown by TREM2+ cells may be involved in proliferation of microglial precursors in the developing brain. It has been previously predicted that microglial proliferation is a physiological mechanism contributing to the acquisition of the adult microglial cell population [4]. Although the expression of TREM2 diminished from P5 onwards, except in svz; CD206, CD86, CD16/32 and MHCII were still present, at least in a subpopulation of microglia. Nevertheless, the functional implication of these changes in the phenotype of TREM2+ cells are not known yet.

Recently, the role of microglia and perinatal inflammation has been associated with certain psychiatric disorders [38], [68], [69], [70] while loss-of-function mutation of TREM2 has been associated with several neurodegenerative disorders or dementia [71], [72], [73], [74]. Therefore, the regional differences in TREM2+ microglia phenotype during postnatal development along with other genetic or environmental factors might be involved in pathological manifestations throughout life. Finally, as microglia share some markers during healthy brain development and after an injury, the understanding on the control of microglial response during development opens new targets for microglial modulation after injury, especially in neonates.

### Conclusions

Microglia are involved not only in monitoring the brain parenchyma, cleaning the cell debris and synaptic contacts overproduced but also in maintaining the brain homeostasis. The TREM2-DAP12 complex is essential for brain as loss-of-function of one of these proteins produces an imbalance in brain homeostasis leading to diseases. This study suggests a putative role in control of spatio-temporal distribution of this complex during postnatal development. We observed that TREM2 was expressed during first week in grey and white matter; however, after this period it was only present in white matter till P10. We also observed phenotypic changes in TREM2+ cells with age and region, suggesting that the microglial population is heterogenic. It is noteworthy that different phenotypes of TREM2+ microglial cells coexist in the same region. The increase of TREM2 in several pathologies may recapitulate their function during postnatal development, so the better understanding of this period can open new pathways for future therapies.

## Supporting Information

Figure S1 Developmental expression of TREM2. (A–D), developmental expression of TREM2 in rostral corpus callosum (ros cc) at P1 (A), P3 (B), P5 (C) and P7 (D), showing no difference in expression pattern. (E–H) Changes in cortex (cx) at P1 (E), P3 (F), P5 (G) and P7 (H), showing a progressive reduction in TREM2 expression from P3. (I–L) TREM2 expression in caudate-putamen (cp) at P1 (I), P3 (J), P5 (K) and P7 (L), showing no changes. (M–P) TREM2 expression in thalamus (tl) at P1 (M), P3 (N), P5 (O) and P7 (P), showing a progressive reduction on TREM2 expression after P3. Scale bar = 50 µm.
(TIF)

Figure S2 TREM2 expression was not observed in oligodendrocytes. Double immunofluorescence was performed for colocalization study of TREM2 (green) with (A) olig2 (red), a pan marker of oligodencrocytes and (B) PDGFRalpha (red), a marker for early oligodendrocyte progenitors. No expression of TREM2 was observed in oligodendrocytes at any time or region studied. DAPI was used for nuclear staining (blue) Scale bar = 20 µm.
(TIF)

Figure S3 Phenotypic characterization of cortical TREM2+ microglia. TREM2 co-expression with: CD206 (A–B), CD16/32 (C–D), CD86 (E–F) and MHCII (G–H) was studied in cortex at P1 (A, C, E and G) and P7 (B, D, F and H). Insets beside each figure represent separated channels: CD206, CD16/32, CD86 and MHCII in red, TREM2 in green and Iba1 in blue. Triple colocalization can be seen in purple. Arrows represent cytoplasmatic expression of MHCII. Cx: cortex; Scale bar for A and B = 50 µm; scale bar for C–H = 20 µm.
(TIF)

Figure S4 Phenotypic characterization of TREM2+ microglia in fimbria. TREM2 co-expression with: CD206 (A–B), CD16/32 (C–D), CD86 (E–F) and MHCII (G–H) was studied at P1 (A, C, E and G) and P7 (B, D, F and H). Insets beside each figure represent separated channels: CD206, CD16/32, CD86 and MHCII in red, TREM2 in green and Iba1 in blue. Triple colocalization can be seen in purple. fim: fimbria. Scale bar for A and B = 50 µm; scale bar for C–H = 20 µm.
(TIF)
