# Supplementary figures and images for: Differential Modulation of TREM2 Protein during Postnatal Brain Development in Mice
Source: PLoS One. 2013 Aug 19;8(8):e72083. doi: 10.1371/journal.pone.0072083 (PMC3747061; doi:10.1371/journal.pone.0072083)

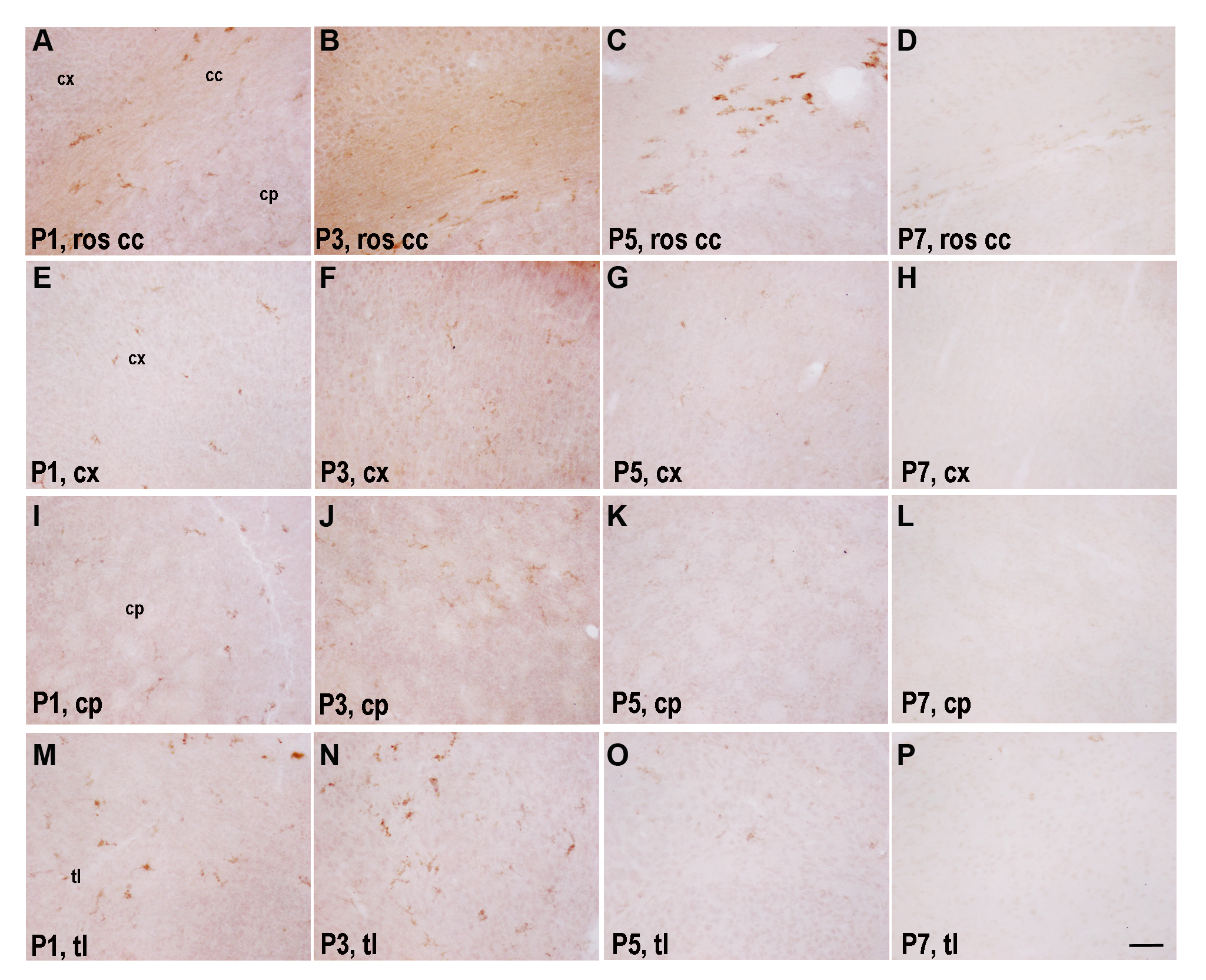

Supplement: Figure S1 — Developmental expression of TREM2. (A–D), developmental expression of TREM2 in rostral corpus callosum (ros cc) at P1 (A), P3 (B), P5 (C) and P7 (D), showing no difference in expression pattern. (E–H) Changes in cortex (cx) at P1 (E), P3 (F), P5 (G) and P7 (H), showing a progressive reduction in TREM2 expression from P3. (I–L) TREM2 expression in caudate-putamen (cp) at P1 (I), P3 (J), P5 (K) and P7 (L), showing no changes. (M–P) TREM2 expression in thalamus (tl) at P1 (M), P3 (N), P5 (O) and P7 (P), showing a progressive reduction on TREM2 expression after P3. Scale bar = 50 µm. (TIF) [file pone.0072083.s001.tif]

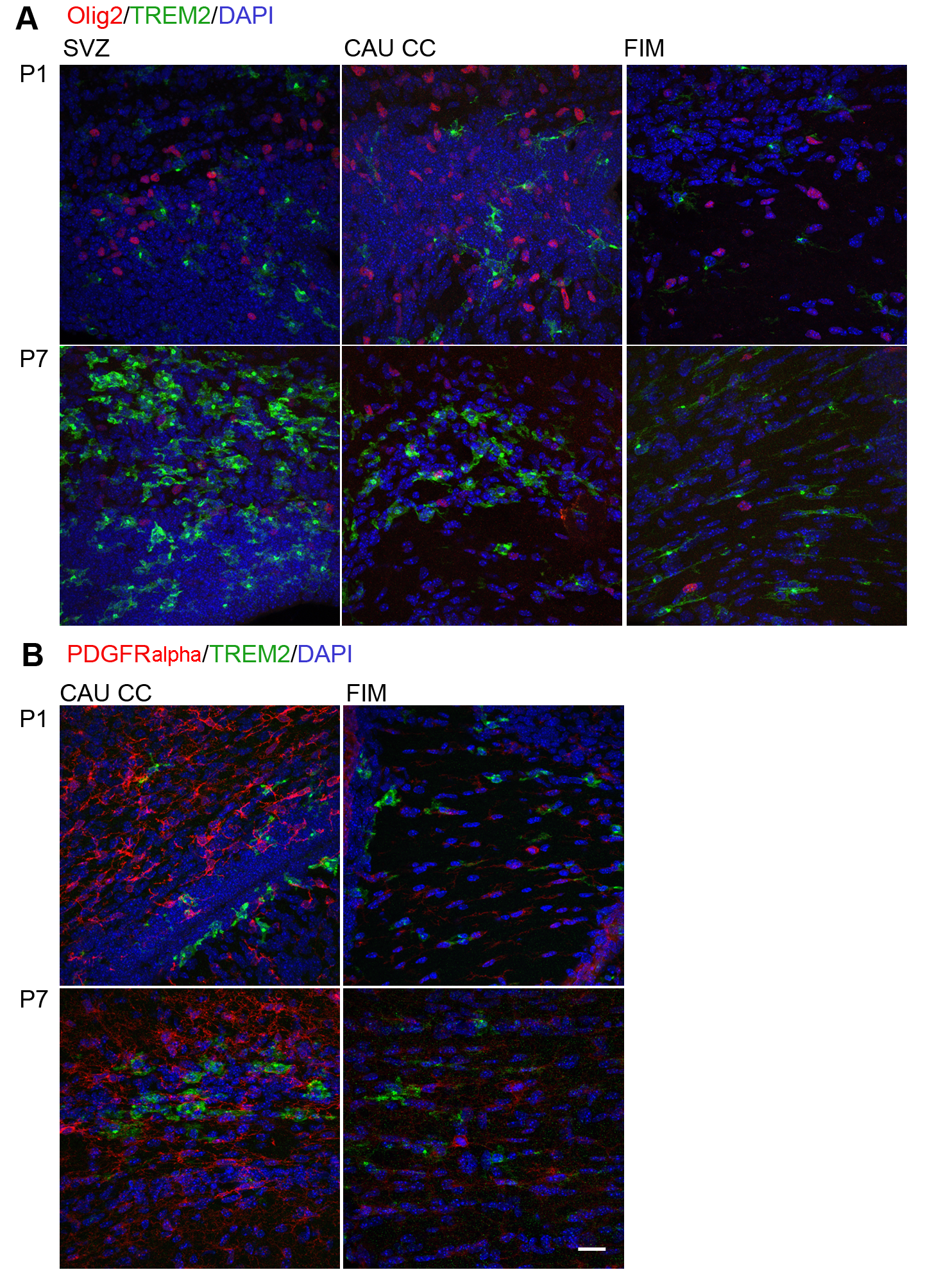

Supplement: Figure S2 — TREM2 expression was not observed in oligodendrocytes. Double immunofluorescence was performed for colocalization study of TREM2 (green) with (A) olig2 (red), a pan marker of oligodencrocytes and (B) PDGFRalpha (red), a marker for early oligodendrocyte progenitors. No expression of TREM2 was observed in oligodendrocytes at any time or region studied. DAPI was used for nuclear staining (blue) Scale bar = 20 µm. (TIF) [file pone.0072083.s002.tif]

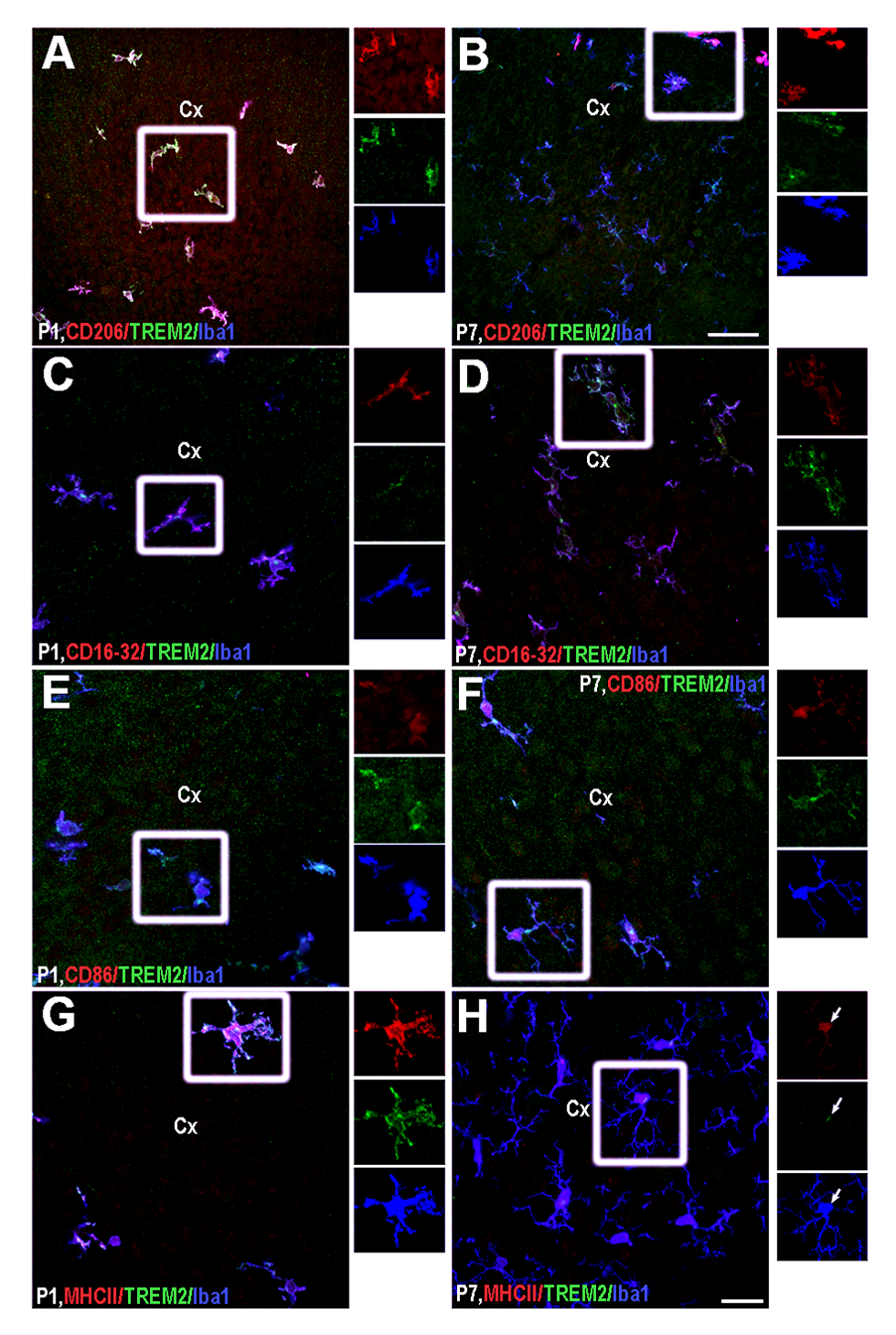

Supplement: Figure S3 — Phenotypic characterization of cortical TREM2+ microglia. TREM2 co-expression with: CD206 (A–B), CD16/32 (C–D), CD86 (E–F) and MHCII (G–H) was studied in cortex at P1 (A, C, E and G) and P7 (B, D, F and H). Insets beside each figure represent separated channels: CD206, CD16/32, CD86 and MHCII in red, TREM2 in green and Iba1 in blue. Triple colocalization can be seen in purple. Arrows represent cytoplasmatic expression of MHCII. Cx: cortex; Scale bar for A and B = 50 µm; scale bar for C–H = 20 µm. (TIF) [file pone.0072083.s003.tif]

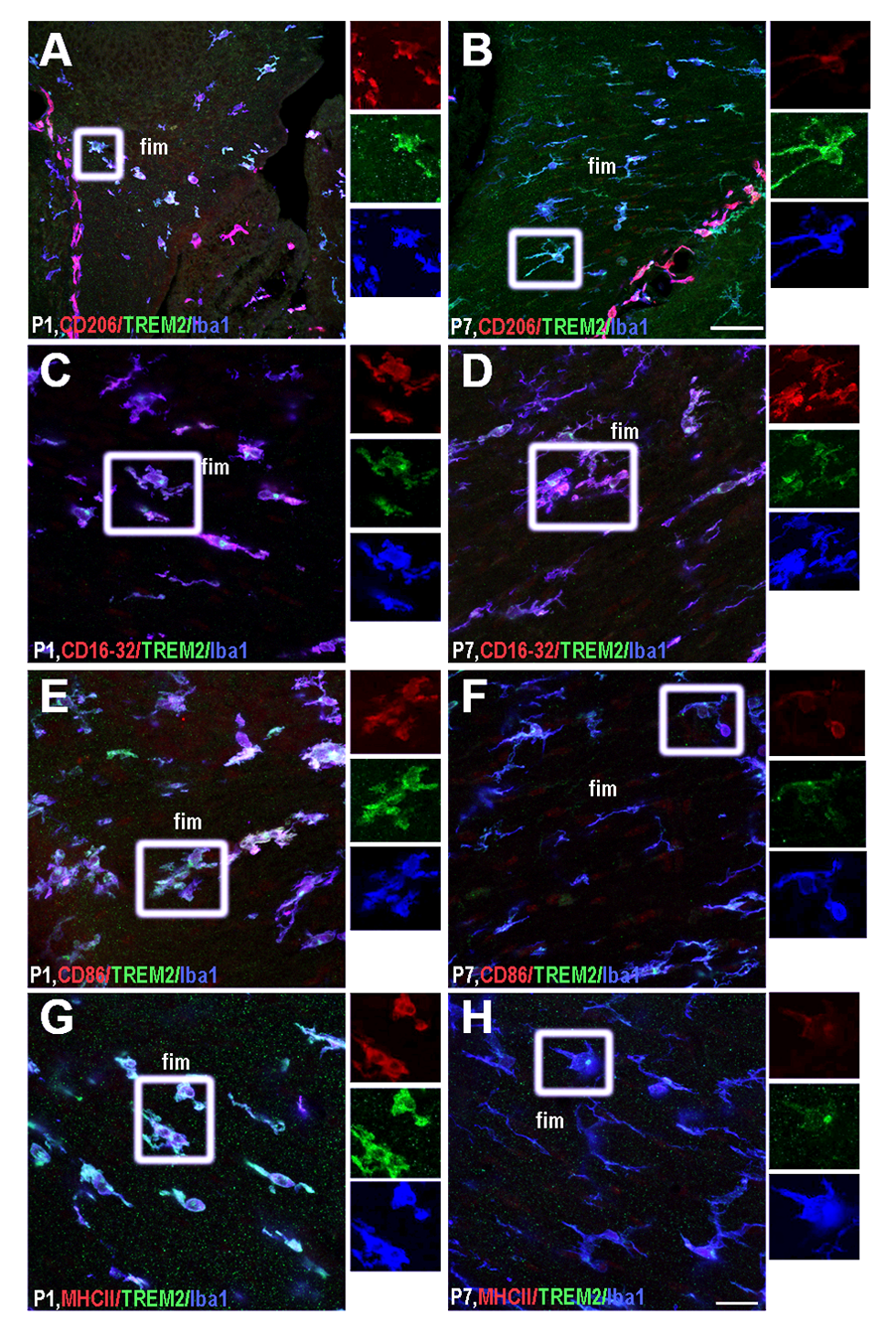

Supplement: Figure S4 — Phenotypic characterization of TREM2+ microglia in fimbria. TREM2 co-expression with: CD206 (A–B), CD16/32 (C–D), CD86 (E–F) and MHCII (G–H) was studied at P1 (A, C, E and G) and P7 (B, D, F and H). Insets beside each figure represent separated channels: CD206, CD16/32, CD86 and MHCII in red, TREM2 in green and Iba1 in blue. Triple colocalization can be seen in purple. fim: fimbria. Scale bar for A and B = 50 µm; scale bar for C–H = 20 µm. (TIF) [file pone.0072083.s004.tif]
